# In-hospital major bleeding in patients with acute coronary syndrome medically treated with dual anti-platelet therapy: Associated factors and impact on mortality

**DOI:** 10.3389/fcvm.2022.878270

**Published:** 2022-10-31

**Authors:** Yihong Sun, Lin Feng, Xian Li, Zhe Wang, Runlin Gao, Yangfeng Wu

**Affiliations:** ^1^China-Japan Friendship Hospital, Beijing, China; ^2^Peking University Clinical Research Institute, Beijing, China; ^3^The George Institute for Global Health at Peking University Health Science Center, Beijing, China; ^4^Faculty of Medicine, University of New South Wales, Sydney, NSW, Australia; ^5^Graduate School of Peking Union Medical College, Chinese Academy of Medical Sciences, Beijing, China; ^6^The Department of Cardiology, Cardiovascular Institute and Fuwai Hospital, Chinese Academy of Medical Sciences and Peking Union Medical College, Beijing, China; ^7^Department of Epidemiology and Biostatistics, Peking University School of Public Health, Beijing, China

**Keywords:** acute coronary syndrome, risk factors, major bleeding, mortality, antithrombotic therapy

## Abstract

**Objective:**

Major bleeding is associated with poor hospital prognosis in patients with acute coronary syndrome (ACS). Despite its clinical importance, there are limited studies on the incidence and risk factors for major bleeding in ACS patients with dual anti-platelet therapy (DAPT) without access to revascularization.

**Methods:**

We analyzed data from 19,186 patients on DAPT after ACS with no access to revascularization from Clinical Pathway for Acute Coronary Syndrome in China Phase 3 (CPACS-3) cohort, which was conducted from 2011 to 2014. Major bleeding included intracranial hemorrhage, clinically significant bleeding, or bleeding requiring blood transfusion. Factors associated with in-hospital major bleeding were assessed using Poisson regressions with generalized estimating equations to account for the clustering effect.

**Results:**

A total of 75 (0.39%) patients experienced major bleeding during hospitalization. Among subtypes of ACS, 0.65% of patients with STEMI, 0.33% with NSTEMI, and 0.13% with unstable angina had in-hospital major bleeding (*p* < 0.001). The patients who experienced major bleeding had a longer length of stay (median 12 vs. 9 days, *p* = 0.011) and a higher all-cause in-hospital death rate (22.7 vs. 3.7%, *p* < 0.001). Multivariable analysis showed advancing age (RR = 1.52 for every 10 years increase, 95% CI: 1.13, 2.05), impaired renal function (RR = 1.79, 95% CI: 1.10, 2.92), use of fibrinolytic drugs (RR = 2.93, 95% CI: 1.55, 5.56), and severe diseases other than cardiovascular and renal diseases (RR = 5.56, 95% CI: 1.10, 28.07) were associated with increased risk of major bleeding, whereas using renin–angiotensin system inhibitors (RR = 0.54, 95% CI: 0.36, 0.81) was associated with decreased risk of major bleeding. These independent factors together showed good predictive accuracy with an AUC of 0.788 (95% CI: 0.734, 0.841).

**Conclusion:**

Among ACS patients on DAPT, advancing age, impaired renal function, thrombolytic treatment, and severe comorbidities were independently associated with a higher risk of in-hospital major bleeding.

## Key messages

### What is already known on this subject?

**▸** Patients with ACS who are managed without revascularization have a higher risk of downstream ischemic and bleeding events compared to ACS patients with revascularization. There are a considerable proportion of ACS patients medically treated, but the predictors and impact of major bleeding in those patients remain unexplored.

### What might this study add?

**▸** The incidence of in-hospital major bleeding is 0.39% and GI bleeding (56 [75.6%]) was the most common type. In multivariable analysis, independent predictors for major bleeding included advancing age, impaired renal function, and severe diseases other than cardiovascular and renal disease. The multivariable model showed good predictive accuracy with an AUC of 0.788 (95% CI: 0.734, 0.841). Patients who suffered major bleeding had a higher risk of in-hospital death.

### How might this impact clinical practice?

**▸** This study suggested that we need to identify and develop risk stratification tools for major bleeding when deciding on the antithrombotic treatment strategy. Procedures for the prevention of major bleeding should be considered in patients with high-bleeding risk.

## Introduction

Advances in the management of acute coronary syndrome (ACS), including the use of dual antiplatelet therapy (DAPT), have been associated with a decline in mortality in patients with or without percutaneous coronary intervention (PCI) (1, 2). Yet, such improvements come at the price of an increased risk of bleeding and associated mortality (3–5). There is ongoing interest in initiatives to reduce bleeding complications in the setting of ACS. To facilitate risk stratification of patients, multiple scoring systems have been developed using heterogeneous cohorts and different definitions (5–8). CRUSADE (Can Rapid Risk Stratification of Unstable Angina Patients Suppress Adverse Outcomes With Early Implementation of the ACC/AHA Guidelines) bleeding risk score is the most established but is limited by weak discriminative ability, especially among patients with ACS managed noninvasively, the elderly, and patients with renal failure (9–11). In the European Society of Cardiology (ESC) guidelines, the CRUSADE score has a class IIb recommendation restricted to patients receiving coronary angiography (12). As a result, ambiguity about how to effectively identify patients at high bleeding risk remains, and it is reflected in the varying inclusion criteria adopted by the completed and ongoing trials in such patients (13–15). Finally, limited data have been published in the contemporary era of increased use of a conservative approach for patients with ACS.

Even though early angiography with the intent to perform revascularization is recommended as the first-line treatment for patients with ACS, a substantial proportion of patients are treated with optimal medical therapy alone because of a high comorbidity burden, extensive coronary artery disease not amenable to revascularization, or lack of flow limiting obstructive coronary artery disease (2, 16). However, patients with ACS who are managed medically without revascularization also have a higher risk of downstream ischemic and bleeding events compared with patients with ACS managed with revascularization. The impact of bleeding on patient outcomes following ACS treated medically with DAPT, but without PCI, remains unexplored (17, 18).

The aim of the present study was to investigate factors associated with in-hospital major bleeding and to evaluate its impact on hospital length of stay and mortality in ACS patients taking DAPT from Chinese hospitals with no PCI capacity.

## Methods

### Study population

The study population was sourced from the Clinical Pathway for Acute Coronary Syndrome in China Phase 3 (CPASC-3) study, which was conducted from 2011 to 2014, and registered on www.clinicaltrials.gov (NCT01398228). The design of the CPACS-3 study has been published elsewhere (19, 20). In brief, the study was a registry-based stepped wedge cluster-randomized trial to evaluate the effectiveness of the quality of care improvement initiative in reducing the risk of in-hospital major cardiovascular events among ACS patients in 101 non-PCI Chinese hospitals. All consecutive patients aged 18 years or older with a final diagnosis of ACS at the time of death or discharge were recruited prospectively in the participating hospitals. Data were collected for each patient using standardized case report forms by trained staff. The comprehensive study intervention includes: establishing a quality of care improvement executive group, implementing the clinical pathways, training all medical staff on ACS management, regular key performance indicators monitoring and feedback, online expert consultations, and patient education. CPACS-3 study was conducted in accordance with the Declaration of Helsinki and Good Clinical Practice guidelines. The Peking University IRB approved the study (IRB00001052-11037). Written consent was acquired from all participants. In the present study, our analyses included only 19,186 patients who received dual antiplatelet therapy during hospitalization (Supplementary Figure 1).

### Data collection

Data collected during hospitalization included socio-demographic characteristics, medical history, type of ACS diagnosis, signs of disease severity at admission, and in-hospital treatments. The plasma lipid profile including total cholesterol (TC), low-density lipoprotein cholesterol (LDL-C), triglyceride (TG), high-density lipoprotein cholesterol (HDL-C), and assays conducted at local laboratories were also collected. The estimated glomerular filtration rate (eGFR) was calculated using the Modification of Diet in Renal Disease (MDRD) equation. The definition of other severe diseases included cancer and other life-threatening disease judged by the investigators, for example, advanced liver disease and sepsis. The definition of impaired renal function is either the patients had a history of chronic kidney disease or eGFR < 60 ml/min/1.73 m^2^. The definition of impaired cardiac function is the history of chronic heart failure or Killip level in II, III, and IV at admission or left ventricular ejection fraction (LVEF) < 50% during the index hospitalization.

### Clinical outcomes

The in-hospital major bleeding was defined as intracranial hemorrhage, clinically significant gastrointestinal (GI) bleeding, or another bleeding that required blood transfusion as described in the protocol. Other bleeding events were not recorded according to the CPACS-3 study protocol. All the clinical events were adjudicated by the independent clinical event committee.

### Statistical analyses

For descriptive purposes, means or medians were calculated to present continuous variables depending on sample distribution and were compared by *t-*test or appropriate nonparametric tests between patients with and without in-hospital major bleeding. Categorical variables were presented as numbers or percentages and differences between groups were tested by the χ^2^ test.

Poisson regression with generalized estimating equations was used to explore the independent predictors of major bleeding during hospitalization, with an exchangeable correlation structure to account for the clustering effect within hospitals. Potential associated factors in multivariable analyses were selected based on clinical interests and the results of univariate analyses (see Supplementary Table 1). The specificity and sensitivity of this model for the prediction of mortality were evaluated by the C-statistic, which is equivalent to the area under the receiver-operating characteristics (ROC) curve.

Multiple imputations with regression method and discriminant function method were used to impute four continuous variables (body mass index, serum creatinine, TC, LDL-C) and three binary variables (troponin positive at presentation, in-hospital heparin/low molecular weight heparin treatment, and pre-hospital delay) correspondingly by fully conditional specification methods. The number of imputed databases was five. The SAS V.9.4 (SAS Institute, Cary, North Carolina, USA) was used to perform all data analyses. The statistical significance level α was set at 0.05.

## Results

### Incidence and types of major bleeding

**Figure 1:**
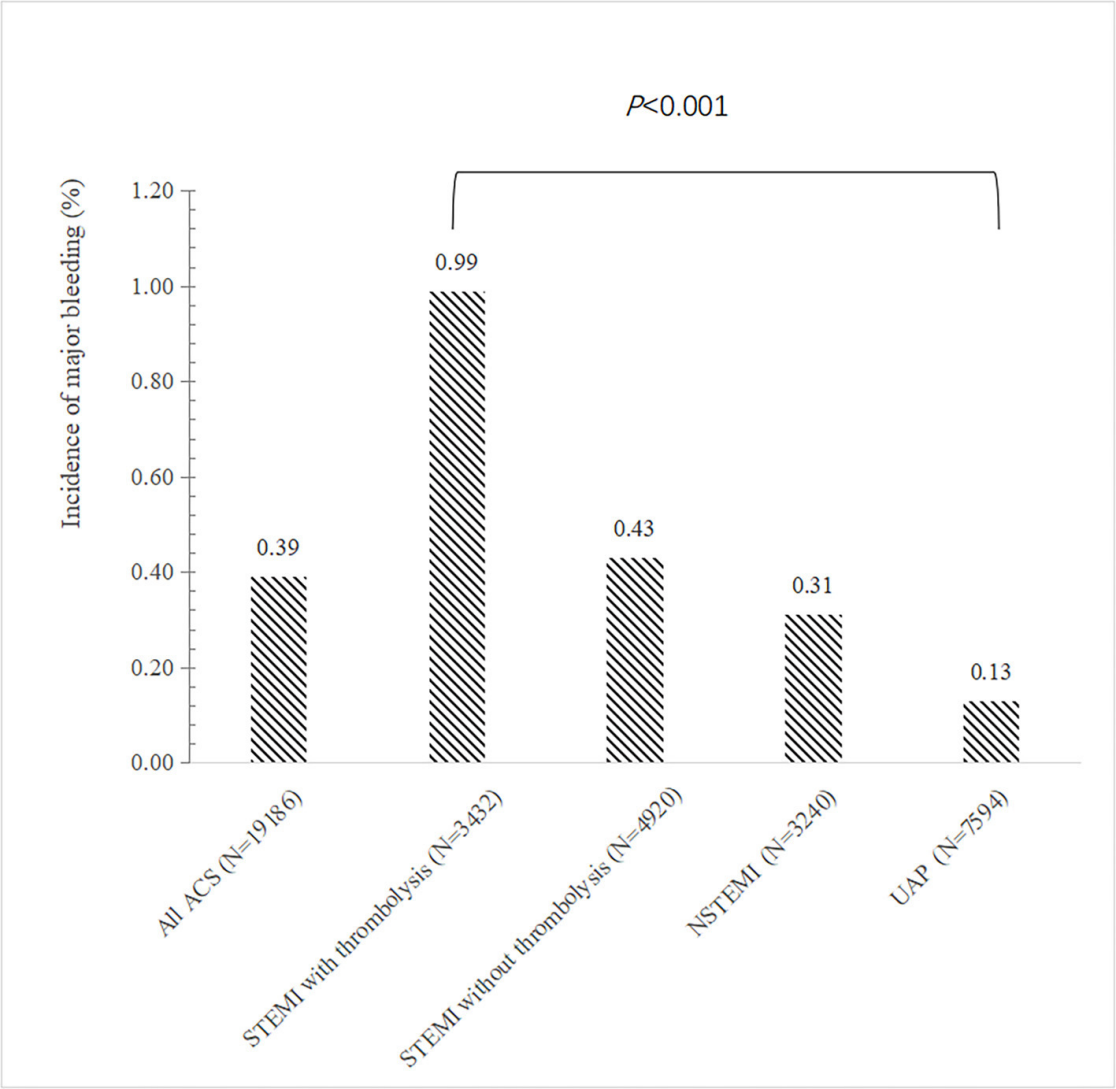
Incidence of major bleeding according to the type of ACS. The bar showed the incidence of in-hospital major bleeding according to the type of ACS. ACS, acute coronary syndrome; STEMI, ST elevation myocardial infarction; NSTEMI, non–ST-segment elevation myocardial; UA, unstable angina.

The overall incidence of major bleeding during hospitalization was 0.39% (75 out of 19,186 patients) in ACS patients on DAPT (Figure 1). Gastrointestinal bleeding accounted for 76% of all major bleeding events. The second most frequent cause of major bleeding was intracranial hemorrhage, which occurred in 11 patients (14.7%) (Supplementary Figure 2).

### Comparison of clinical characteristics between patients with and without major bleeding

Clinical characteristics of patients with and without in-hospital major bleeding are shown in Table 1. Compared with patients without major bleeding, patients with major bleeding were older, more likely to have a prior history of stroke/transient ischemic attack (TIA), chronic kidney disease, and severe diseases other than cardiovascular and renal diseases, had less pre-hospital delay but presented more seriously on admission, e.g., impaired renal and cardiac function, and were more likely to be STEMI patients and receive thrombolysis. Interestingly, patients with major bleeding were less frequently receiving ACEI/ARB and β-blockers.

**Table 1:** Baseline characteristics.

	**No major bleeding** ** (*N =* 19,111)**	**Major bleeding** ** (*N =* 75)**	***P* value**
Age (year), median (Q1, Q3)	64 (56,72)	70 (63,75)	< 0.001
Male, *n* (%)	12,329 (64.5)	47 (62.7)	0.739
Current Smoker, *n* (%)	5,444 (28.5)	20 (26.7)	0.728
BMI (kg/m2), median (Q1, Q3)	23.7 (12.9,25.7)	23.7 (21.5,24.3)	0.266
**Medical history**			
Diabetes, *n* (%)	2,617 (13.7)	8 (10.7)	0.447
Hypertension, *n* (%)	9,070 (47.5)	37 (49.3)	0.746
Dyslipidemia, *n* (%)	897 (4.7)	3 (4.0)	0.777
Prior Angina, *n* (%)	3,980 (20.8)	12 (16.0)	0.304
Prior MI, *n* (%)	1,653 (8.6)	5 (6.7)	0.542
Congestive heart failure, *n* (%)	737 (3.9)	6 (8.0)	0.063
Prior Stroke/TIA, *n* (%)	1,694 (8.9)	14 (18.7)	0.003
Chronic kidney disease, *n* (%)	154 (0.9)	3 (4.1)	0.003
Other severe disease, *n* (%)	82 (0.4)	2 (2.7)	0.003
Pre-hospital delay >6 hour, *n* (%)	7,577 (49.0)	18 (30.5)	0.004
**Clinical presentation at admission**			
CPR, *n* (%)	614 (3.3)	8 (11.0)	< 0.001
SBP, (mmHg), median (Q1, Q3)	131 (120, 150)	135 (116, 150)	0.540
DBP, (mmHg), median (Q1, Q3)	80 (70, 90)	80 (70, 90)	0.300
HR, (beats/min), median (Q1, Q3)	75 (64, 86)	76 (65, 92)	0.481
Serum creatinine, (μmol/L), median (Q1, Q3)	75 (62, 91)	89 (79,125)	< 0.001
eGFR (%), median (Q1, Q3)	96 (75, 119)	75 (52, 99)	< 0.001
< = 60%, *n* (%)	1,996 (12, 3)	22 (31.9)	< 0.001
61%-89%, *n* (%)	4,970 (30.6)	26 (37.7)	
≥90%, *n* (%)	9,293 (57.2)	21 (30.4)	
Troponin positive, *n* (%)	7,085 (50.0)	39 (58.2)	0.181
TC, (mmol/L), median (Q1, Q3)	4.5 (3.8, 5.2)	4.2 (3.6, 5.0)	0.148
LDL-C, (mmol/L), median (Q1, Q3)	2.6 (2.1, 3.2)	2.6 (2.0,3.0)	0.435
Killip II-IV, *n* (%)^#^	3,013 (33.1)	23 (41.1)	0.209
LVEF (%), median (Q1, Q3)	58 (51, 65)	55 (41, 60)	0.045
Impaired heart function, *n* (%)	5,124 (26.8)	33 (44.0)	< 0.001
Impaired renal function, *n* (%)	2,071 (11.0)	22 (29.7)	< 0.001
**Diagnosis**			
STEMI, *n* (%)	8209 (43.0)	54 (72.0)	< 0.001
NSTEMI, *n* (%)	3292 (17.2)	11 (14.7)	
UAP, *n* (%)	7610 (39.8)	10 (13.3)	
**In-hospital medical treatment**			
**DAPT loading statuses**			
No loading, *n* (%)	6,911 (36.2)	19 (25.3)	0.127
Single loading, *n* (%)	2,989 (15.6)	12 (16.0)	
Dual loading, *n* (%)	9,211 (48.2)	44 (58.7)	
Heparin/LMWH, *n* (%)	16,033 (90.9)	64 (94.2)	0.351
Statin, *n* (%)	18,412 (96.4)	72 (96.0)	0.869
ACEI/ARB, *n* (%)	12,178 (63.7)	35 (46.7)	0.002
CCB, *n* (%)	2,794 (15.0)	8 (11.1)	0.357
Beta-blocker, *n* (%)	13,254 (69.5)	40 (53.3)	0.003
Thrombolysis*, *n* (%)	3,310 (40.3)	33 (61.1)	0.002

#, among MI patients (*N* = 11,566); *, among STEMI patients (*N* = 8,263).

BMI, body mass index; TIA, transit ischemic attack; HR, heart rate; SBP, systolic blood pressure; DBP, diastolic blood pressure; CPR, cardiopulmonary resuscitation;TC, total cholesterol; LDL-C, low-density lipoprotein cholesterol; LVEF, left ventricular ejection fraction; STEMI, ST elevation myocardial infarction; NSTEMI, non–ST-segment elevation myocardial; UA, unstable angina; DAPT, dual antiplatelet therapy; ACS, acute coronary syndrome; LMWH, low molecular weight heparin; ACEI, angiotensin-converting enzyme inhibitors; ARB, angiotensin receptor blockers; CCB, Calcium Channel Blockers; eGFR, estimated glomerular filtration rate.

We also compared the baseline characters between patients with and without ACEI/ARB treatment as shown in Supplementary Table 2. Compared to patients given ACEI/ARB, those without ACEI/ARB treatment had high-risk clinical features on admission, including more CPR performed, lower blood pressure, and worse heart function, although they were less likely to have a history of hypertension, diabetes, heart failure, and stroke. And patients with STEMI were less likely to receive ACEI/ARB treatment compared to patients with NSTEMI and UA.

### The independent predictors for in-hospital major bleeding

**Figure 2:**
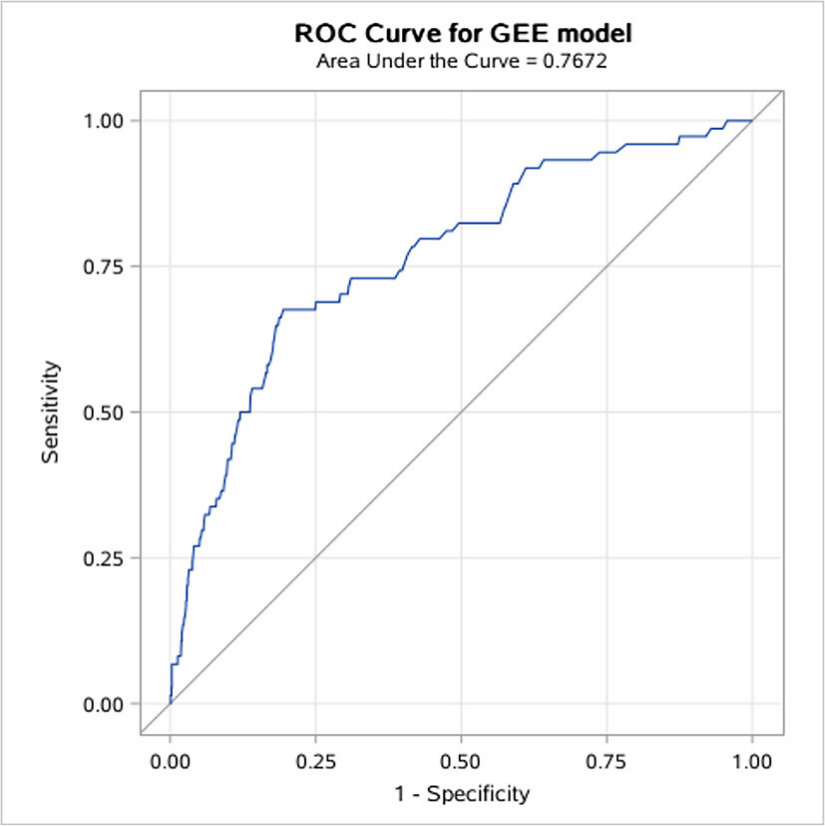
ROC curve for in-hospital bleeding.

STEMI patients who received thrombolytic therapy had the highest risk of major bleeding compared to other types of ACS, while patients with unstable angina were less likely to bleed (Figure 1). In multivariable regression analysis, we found that the higher risk of major bleeding was significantly associated with advancing age, impaired renal function, receiving thrombolytic therapy, history of severe disease other than a cardiovascular and renal disease, and less use of ACEI/ARB during hospitalization (Table 2). The AUC of this predictive model for in-hospital bleeding is 0.788 (95% CI: 0.734, 0.841) (Figure 2). We also compared the performance of our model with the CRUSADE score using ROC analysis in 9,084 patients. The AUC of our model is higher than the CRUSADE score (Supplementary Figure 3).

**Table 2:** Risk factors associated with in-hospital major bleeding by multivariable regression analysis.

**Parameters**	**RR**	**95% CI**		**P-value**
Age, every 10 years increased	1.52	1.13	2.05	0.006
Female	1.00	0.53	1.88	0.997
**BMI (kg/m** ^**2**^ **)**				
< 22	1.08	0.33	3.46	0.894
22–23.6 (reference)	1.00			.
23.7–25.7	1.46	0.58	3.67	0.412
> =25.8	0.91	0.24	3.48	0.883
Current smoker	1.19	0.56	2.54	0.646
Hypertension history	1.20	0.73	1.96	0.471
Diabetes mellitus history	0.80	0.38	1.68	0.555
Dyslipidemia history	1.02	0.31	3.40	0.970
Stroke/TIA history	1.92	0.97	3.82	0.062
Other severe disease	5.56	1.10	28.07	0.038
CPR or SB*P < * 90mmHg or HR>100bpm	0.99	0.51	1.92	0.972
**SBP**				
< 100 mmHg	0.81	0.32	2.07	0.656
100–119 mmHg	0.69	0.32	1.48	0.342
140–159 mmHg	1.17	0.68	2.01	0.580
≥160 mmHg	1.10	0.60	2.03	0.761
**HR**				
< 65 bpm	1.02	0.41	2.52	0.962
65–74 bpm	1.69	0.90	3.16	0.101
75–84 bpm	1.00			
≥85 bpm	1.70	0.80	3.63	0.171
Impaired cardiac function	1.52	0.89	2.59	0.123
Impaired renal function	1.79	1.10	2.92	0.019
Troponin positive	0.89	0.54	1.48	0.655
**Diagnosis**				
STEMI	2.17	0.96	4.92	0.062
NSTEMI	1.65	0.73	3.71	0.228
UA	1.00			
**DAPT loading statuses**				
No loading	1.00			
Only one loading	0.93	0.36	2.45	0.889
Dual loading	1.31	0.72	2.41	0.376
Thrombolysis	2.93	1.55	5.56	0.001
Heparin/LMWH	1.02	0.34	3.07	0.967
β-blocker	0.67	0.38	1.20	0.179
ACEI/ARB	0.54	0.36	0.81	0.003
CCB	1.06	0.47	2.38	0.884
Statin	1.06	0.36	3.17	0.912

CI, confidence interval; BMI, body mass index; TIA, transient ischemic attack; HR, heart rate; SBP, systolic blood pressure; DBP, diastolic blood pressure; CPR, cardiopulmonary resuscitation; TC, total cholesterol; LDL-C, low-density lipoprotein cholesterol; LVEF, left ventricular ejection fraction; STEMI, ST elevation myocardial infarction; NSTEMI, non–ST-segment elevation myocardial; UA, unstable angina; DAPT, dual antiplatelet therapy; LMWH, low molecular weight heparin; ACEI, angiotensin-converting enzyme inhibitors; ARB, angiotensin receptor blockers; CCB, Calcium Channel Blockers; eGFR, estimated glomerular filtration rate.

### The impact of major bleeding on clinical outcomes

**Figure 3:**
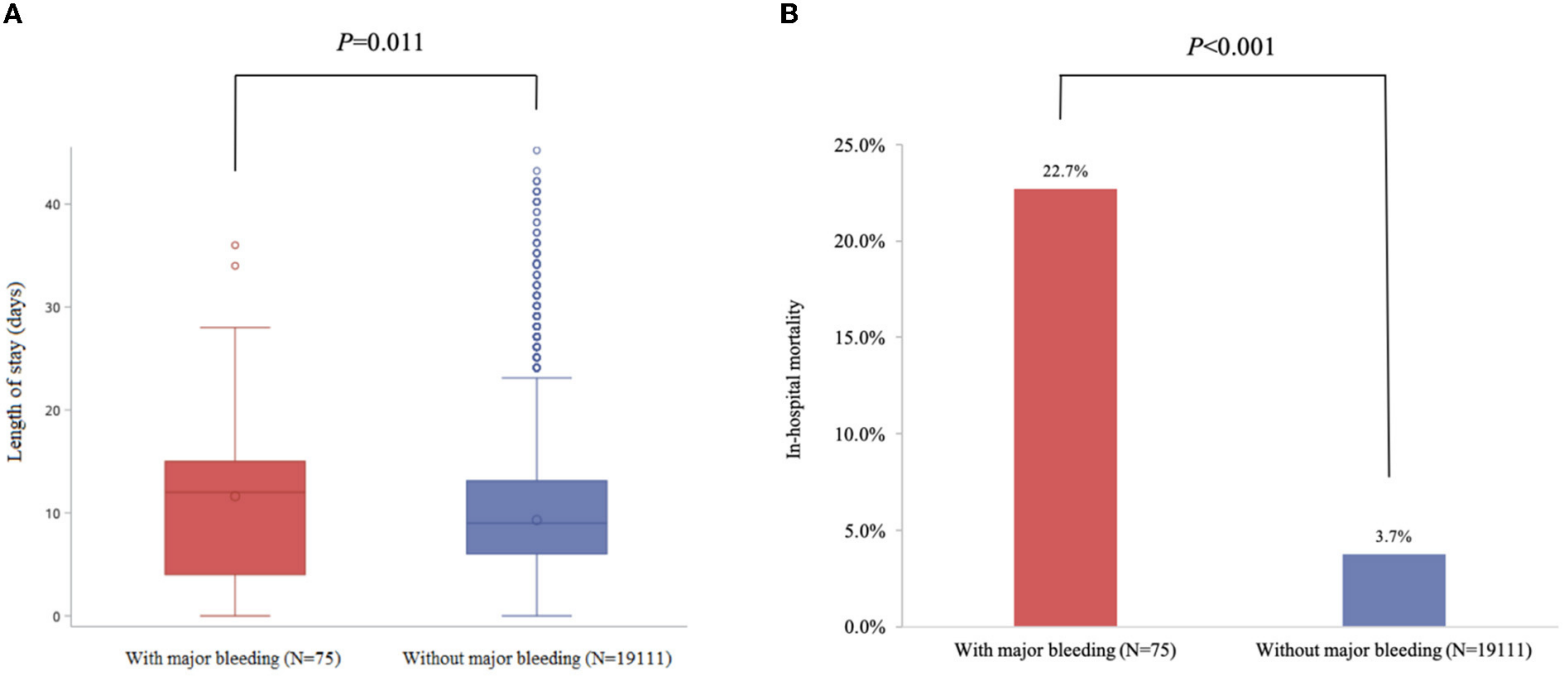
Major bleeding was significantly associated with all-cause in-hospital mortality and length of stay in patients with ACS. **(A)** Length of stay (days). **(B)** in-hospital mortality.

The median time from admission to discharge in patients with major bleeding was 3 days longer than those without major bleeding [9 days Q1–Q3 (6–13) vs. 12.0 days Q1–Q3 (4–15), *p* = 0.011]. The risk of all-cause in-hospital death was significantly higher in patients with major bleeding compared to those without (22.7 vs. 3.7%, *p* < 0.001) (Figure 3).

## Discussion

In this *post-hoc* analysis of a large cluster-randomized trial, the incidence of major bleeding was 0.39% in ACS patients receiving DAPT, which differed significantly from the subtype of ACS, with 0.65% in STEMI patients, 0.33% in NSTEMI patients, and 0.13% in unstable angina patients. Gastrointestinal bleeding was the most common major bleeding during hospitalization. Our multiple regression analysis found that advanced age, impaired renal function, and thrombolytic treatment were the major predictors of the risk of major bleeding. We found that patients not receiving ACEI/ARB and having non-cardiovascular non-renal severe comorbidities, such as cancer might carry a greater risk of bleeding, which has not been previously reported.

Hemorrhagic complications occur with a frequency of 1–10% during treatment for ACS and/or after PCI (12–24). The incidence of major bleeding in our study was low (0.39%) compared with ACTION (10.8%), ACUITY (7.3% in 30 days), and CRUSADE (9.4%), which also enrolled patients with ACS (5, 21, 23). However, the incidence was comparable with those in the contemporary treatment, such as the rate of the thrombolysis in myocardial infarction (TIMI) major bleeding was about 0.9% in the ACCOST trial (24). The rate of bleeding reported in a Chinese cohort of acute myocardial infarction was 0.53% (25). This wide variability in the measured incidence could be due to multiple reasons, including differences in definitions of bleeding across data sets, differences in patient characteristics, and differences in concomitant therapies. In the ACTION registry (21), the definition of major bleeding included an absolute Hb decrease of 4 g/dl, intracranial hemorrhage, documented or suspected retroperitoneal bleed, any red cell blood transfusion with baseline Hb of 9 g/dl, or any red cell transfusion with Hb 9 g/dl and a suspected bleeding event. Our bleeding definition included only non–coronary artery bypass graft-related bleeds that were intracranial or GI bleeding leading to a blood transfusion during a short observation period. In the SWEDENHEART registry, the rate of major bleeding was 1.4% in patients with AMI, of which the patients were 5 years older than this cohort and 50% received PCI (22). The bleeding risk of patients included in the ACCOAST trial was not as high as patients in this study, but all received a more potent P2Y12 inhibitor (prasugrel) (24). In addition, the rate of loading of dual antiplatelet therapy was no more than 50% and there were neither use of potent P2Y12 antagonists nor glycoprotein IIb/IIIa inhibitor in our study patients. In our study, treatment without revascularization and avoiding bleeding in the vascular access site may lead to a lower rate of in-hospital bleeding, although major bleeding in the vascular access site is uncommon.

Previous studies in patients with ACS have demonstrated a greater risk of in-hospital bleeding in those with renal dysfunction (26). This association of bleeding with renal dysfunction has been attributed to several mechanisms: activation of the endogenous fibrinolytic system; impaired platelet function; prolongation of half-life and consequent accumulation of antithrombotic agents. The studies reporting the association between renal dysfunction and risk of bleeding events after ACS were mostly in patients receiving an invasive treatment strategy (27), whereas the evidence was very limited in patients without revascularization. Our study found that impaired renal function was an independent predictor of the risk of major bleeding in ACS patients with a conservative treatment strategy. This finding also explained why the sex difference in risk of bleeding could disappear after adjustment for baseline creatinine and other confounders, as reported by Chandiramani et al. (28).

To our surprise, we found that the use of ACEI/ARB was associated with a lower risk of major bleeding in patients with ACS on DAPT. It was also reported to be associated with less bleeding risk in patients receiving left ventricular assist devices, previously (29). Although the exact mechanism by which it occurs is not completely understood, studies have shown that activation of the angiotensin II receptor results in angiogenesis through triggering and augmentation of TGF-β and angiopoietin-2 (Ang-2) pathways (30). We could not propose the mechanism of the protective effects of ACEI/ARB in ACS patients medically treated. The apparent benefit of ACEI/ARB and beta-blocker treatment in preventing major bleeding could well represent a selection bias, with sicker patients at a higher risk of bleeding not given those drugs.

Our study found cancer and other non-cardiovascular non-renal life-threatening disease was associated with in-hospital major bleeding. Malignancy was an exclusion criterion in previous studies, such as the CRUSADE (Can rapid risk stratification of unstable angina patients suppress adverse outcomes with early implementation of the ACC/AHA guidelines), DAPT (Dual antiplatelet therapy study), and Trilogy-ACS (Targeted platelet inhibition to clarify the optimal strategy to medically manage acute coronary syndromes) (5, 31, 32). Cancers that are systemic, such as those emanating from the gastroesophageal tract, are more likely to increase bleeding complications. Therefore, thoughtful consideration should be given to this group of patients when deciding on a medication management strategy after ACS to minimize the risk of bleeding.

The application of bleeding risk stratification is an integral part of the management of patients presenting with ACS. Several bleeding risk scores have been recommended to guide the long-term duration of DPAT (6, 32). While there is limited data to support the decision on optimal antithrombotic treatment in the acute phase of ACS. Studies have shown that more potent P2Y12, prasugrel, or ticagrelor, is associated with a higher risk of bleeding (33). Lv et al. found that the AUC for the CRUSADE score was 0.693 in patients taking antiplatelet drugs, which is much lower than the (ABC)2D score with an AUC of 0.857 (34). In elderly patients with PCI, hemoglobin discrimination ability was not inferior to that of the bleeding risk scores, with AUCs of 0.673, 0.666, and 0.600 for hemoglobin, PRECISE-DAPT, and CRUSADE, respectively (11). The Academic Research Consortium (ARC) recently proposed a list of clinical criteria to define patients at high bleeding risk (HBR) (35). This risk model has been recently validated in patients with PCI (36, 37). However, bleeding risk assessment based on ARC-HBR criteria may be difficult to apply in routine clinical practice as several of the criteria are quite detailed, which included information on comorbidities, bleeding history, iatrogenic factors, and laboratory parameters. The predictive value of these scores has not been established in medically treated patients with ACS.

In the analysis of the PLATO trial, ticagrelor compared with clopidogrel was associated with similar total major bleeding but increased non-CABG and non-procedure-related major bleeding, primarily after 30 days of study drug treatment (2). In the ACUITY trial, the upstream antithrombotic treatment before PCI was associated with excess bleeding with mortality implications in NSTE-ACS patients (38). Whether patients with high bleeding risk could benefit from optimal use of P2Y12 inhibitors based on the bleeding risk model need prospective studies. Until now, none of the risk prediction scores have been prospectively evaluated in randomized clinical trials, therefore the value of improving patient outcomes remains unclear.

Antithrombotic therapy should be balanced between ischemic and bleeding risks during hospitalization. Several studies have demonstrated that bleeding is associated with an increased risk for short- and long-term adverse outcomes, including death, nonfatal MI, stroke, and stent thrombosis (3, 4, 33, 34). The exact mechanisms underlying this relationship are not known, but may include the cessation of evidence-based therapies, including antiplatelet agents, ß-blockers, and/or statin therapies in patients who suffer bleeding complications, the direct effects of blood transfusion used to treat bleeding, or greater prevalence of comorbidities in patients who bleed, as well as a deleterious role of anemia.

### Limitation

This is a *post-hoc* analysis from a prospective randomized trial, and the results should be considered only hypothesis generating. This study was unique because all types of patients with ACS were enrolled, which included STEMI patients receiving thrombolytic therapy. However, the findings of this study should be interpreted in light of some limitations. First, as per *post-hoc* analysis, some important information on the prediction of bleeding risk was not collected in the original study, such as previous bleeding history, platelet account, anemia, and baseline hemoglobin. Therefore, we could not validate the bleeding scores recommended by guidelines. The cause of death was not adjudicated for the identification of bleeding-related deaths. Second, the limited number of bleeding events could lead to the generalization of the results. Furthermore, the rate of bleeding could be underestimated as we did not collect the changes in hemoglobin. That might be also one of the reasons leading to the low incidence of major bleeding reported in this study. Third, the missing values of a few collected variables were high. We used multiple imputations to impute them by creating specific imputation equations for each variable and combined estimates from five imputed databases. Third, our findings may not be generalizable to all patients with ACS at present, because patients enrolled in our trial were managed without revascularization and the newer oral antiplatelet drugs (like prasugrel and ticagrelor) were not available during the study period. Bleeding related to invasive procedures has been decreasing because of the use of radial routes and newer oral anticoagulants. However, findings from our study may still have clinical significance since primary PCI remains inaccessible to a large population of patients.

## Conclusion

In summary, major bleeding during hospitalization was found to be significantly associated with in-hospital mortality. Patients treated with DAPT who experienced major bleeding had distinct characteristics. Identification of these characteristics and the development of a risk stratification tool are important for deciding on optimal antithrombotic therapy after ACS.

## Data availability statement

The original contributions presented in the study are included in the article/Supplementary material, further inquiries can be directed to the corresponding author/s.

## Ethics statement

The studies involving human participants were reviewed and approved by the Ethics Committee of the Peking University IRB. The patients/participants provided their written informed consent to participate in this study.

## Author contributions

Conception of design of the work: YS, RG, and YW. Data analysis and interpretation: YS, LF, and XL. YS and YW are responsible for the overall content as guarantors. Drafting of the manuscript and critical revision of the manuscript: All authors. All authors provided final approval of the manuscript.

## Funding

Sanofi, China provided funding to support the original studies of CPACS-3.

## Conflict of interest

The authors declare that the research was conducted in the absence of any commercial or financial relationships that could be construed as a potential conflict of interest.

## Publisher's note

All claims expressed in this article are solely those of the authors and do not necessarily represent those of their affiliated organizations, or those of the publisher, the editors and the reviewers. Any product that may be evaluated in this article, or claim that may be made by its manufacturer, is not guaranteed or endorsed by the publisher.
