# Supplementary material for: In-hospital major bleeding in patients with acute coronary syndrome medically treated with dual anti-platelet therapy: Associated factors and impact on mortality
Source: Front Cardiovasc Med. 2022 Oct 31;9:878270. doi: 10.3389/fcvm.2022.878270 (PMC9661195; doi:10.3389/fcvm.2022.878270)

**Supplement tables and figures**

**Supplement Table 1.** Predicting factors for major bleeding during hospitalization in patients with ACS by univariate analyses.

| Parameters | Major bleeding events | | | |
| --- | --- | --- | --- | --- |
|  | RR | 95% CI | | P value |
| Age, every 10 years | 1.53 | 1.26 | 1.85 | <0.001 |
| Female | 1.10 | 0.62 | 1.94 | 0.741 |
| Current Smoker | 0.85 | 0.45 | 1.62 | 0.626 |
| BMI |  | | | |
| <22 (kg/m2) | 1.26 | 0.40 | 3.91 | 0.667 |
| 22-23.6 (kg/m2) | reference | | |  |
| 23.7-25.7 (kg/m2) | 1.39 | 0.56 | 3.42 | 0.467 |
| ≧25.8 (kg/m2) | 0.75 | 0.20 | 2.87 | 0.649 |
| Medical history |  | | | |
| DM | 0.77 | 0.36 | 1.64 | 0.495 |
| Hypertension | 1.08 | 0.70 | 1.68 | 0.725 |
| Dyslipidemia | 0.82 | 0.24 | 2.84 | 0.723 |
| Angina | 0.71 | 0.39 | 1.27 | 0.244 |
| MI | 0.78 | 0.33 | 1.85 | 0.570 |
| Heart failure | 2.28 | 1.11 | 4.65 | 0.024 |
| Stroke/TIA | 2.36 | 1.30 | 4.36 | 0.006 |
| CKD | 5.06 | 1.63 | 15.66 | 0.005 |
| Other severe disease | 5.88 | 1.25 | 27.70 | 0.025 |
| **Clinical presentation at admission** | | | | |
| CPR | 3.57 | 1.78 | 7.15 | <0.001 |
| SBP |  |  |  |  |
| <100mmHg | 2.08 | 1.03 | 4.20 | 0.040 |
| 100-119 mmHg | 0.91 | 0.45 | 1.86 | 0.799 |
| 120-139 mmHg | reference | | |  |
| 140-159 mmHg | 1.11 | 0.63 | 1.98 | 0.711 |
| ≧160mmHg | 1.05 | 0.56 | 1.99 | 0.877 |
| HR |  |  |  |  |
| <65 bpm | 1.45 | 0.66 | 3.18 | 0.353 |
| 65-74 bpm | 1.44 | 0.76 | 2.74 | 0.267 |
| 75-84 bpm | reference | | |  |
| ≧85 bpm | 1.90 | 1.02 | 3.57 | 0.044 |
| CPR or SBP<90mmHg or HRT>100bpm | 1.81 | 1.11 | 2.95 | 0.017 |
| eGFR |  | | | |
| ≦60% | 4.23 | 2.46 | 7.29 | <0.001 |
| 61%-89% | 2.34 | 1.38 | 3.96 | 0.002 |
| ≧90% | reference | | |  |
| Troponin increased | 1.36 | 0.85 | 2.17 | 0.196 |
| Impaired cardiac function | 2.18 | 1.39 | 3.43 | <0.001 |
| Impaired renal function | 2.81 | 1.73 | 4.57 | <0.001 |
| TC |  |  |  |  |
| <3.8 mmol/L | 1.58 | 0.66 | 3.76 | 0.295 |
| 3.8-4.4 mmol/L | 1.87 | 0.87 | 4.04 | 0.109 |
| 4.5-5.1 mmol/L | reference | | |  |
| ≧5.2 mmol/L | 1.19 | 0.49 | 2.92 | 0.696 |
| LDL-C |  | | | |
| <2.1 mmol/L | 1.38 | 0.66 | 2.89 | 0.391 |
| 2.1-2.5 mmol/L | 1.27 | 0.62 | 2.60 | 0.519 |
| 2.6-3.1 mmol/L | 1.20 | 0.56 | 2.56 | 0.636 |
| ≧3.2 mmol/L | reference | | |  |
| Diagnosis, % |  |  |  |  |
| STEMI | 5.00 | 2.71 | 9.21 | <0.001 |
| NSTEMI | 2.68 | 1.29 | 5.58 | 0.008 |
| UA | reference | | |  |
| **In-hospital medical treatment** |  | | | |
| DAPT loading statuses |  | | | |
| No loading | reference | | |  |
| Only one loading | 1.37 | 0.65 | 2.92 | 0.407 |
| Dual loading | 1.63 | 0.95 | 2.79 | 0.077 |
| Fibrinolysis | 3.77 | 2.50 | 5.67 | <0.001 |
| Heparin/LMWH | 1.18 | 0.44 | 3.19 | 0.743 |
| Statin | 0.83 | 0.28 | 2.45 | 0.736 |
| ACEI/ARB | 0.51 | 0.34 | 0.76 | 0.001 |
| Calciam blocke | 0.70 | 0.35 | 1.43 | 0.333 |
| Beta-blocker | 0.50 | 0.32 | 0.79 | 0.003 |

BMI = body mass index; TIA= transient ischemic attack; SBP=systolic blood pressure; DBP=diastolic blood pressure; CPR= cardiaopulmonary resuscitation ;TC= total cholesterol; LDL-C = low-density lipoprotein cholesterol; LVEF = left ventricular ejection fraction; STEMI = ST elevation myocardial infarction; NSTEMI = non–ST-segment elevation myocardial; DAPT = dual antiplatelet therapy; LMWH = low molecular weight heparin ; ACEI = angiotensin-converting enzyme inhibitors; ARB = angiotensin receptor blockers; eGFR = estimated glomerular filtration rate.

**Table S2 Comparison of the baseline characteristic between patients with and without ACEI/ARB treatment**

|  | With ACEI/ARB (N=12213) | Without ACEI/ARB (N=6973) | P value |
| --- | --- | --- | --- |
| Bleeding events | 35 (0.29) | 40 (0.57) | <0.001 |
| Male | 7713 (63.2) | 4663 (66.9) | <0.001 |
| Age (year), median (Q1, Q3) | 64 (56,72) | 64 (55, 72) | 0.130 |
| Medical history |  |  |  |
| Hypertension | 6743 (55.2) | 2364 (33.9) | <0.001 |
| Diabetes | 1774 (14.5) | 851 (12.2) | <0.001 |
| Dyslipidemia | 628 (5.1) | 272 (3.9) | <0.001 |
| Prior Angina | 2804 (23.0) | 1188 (17.0) | <0.001 |
| Prior MI | 1059 (8.7) | 599 (8.6) | 0.848 |
| Congestive heart failure | 540 (4.4) | 203 (2.9) | <0.001 |
| Prior Stroke/TIA | 1192 (9.8) | 516 (7.4) | <0.001 |
| Chronic kidney disease | 98 (0.9) | 59 (0.9) | 0.815 |
| Other severe disease | 47 (0.4) | 37 (0.5) | 0.141 |
| Clinical presentation at admission |  |  | <0.001 |
| CPR | 245 (2.1) | 377 (5.5) | <0.001 |
| SBP, (mmHg), median (Q1, Q3) | 140 (120,160) | 124 (110,140) | <0.001 |
| DBP, (mmHg), median (Q1, Q3) | 82 (75,95) | 80 (70,88) | <0.001 |
| HR, (beats/min), median (Q1, Q3) | 76 (66,86) | 73 (64,84) | <0.001 |
| Serum creatinine, (μmol/L), median (Q1, Q3) | 75.0 (62.0,91.0) | 74.8 (62.0,92.0) | 0.774 |
| eGFR (%), median (Q1, Q3) | 95.3 (74.5,118.4) | 96.4 (74.6,120.6) | 0.100 |
| <= 60% | 1291 (12.2) | 727 (12.6) | 0.758 |
| 61%-89% | 3282 (31.0) | 1714 (29.8) |  |
| >=90% | 6004 (56.8) | 3310 (57.6) |  |
| Troponin positive | 4593 (49.1) | 2531 (51.9) | 0.002 |
| KillipⅡ-IV# | 1813 (31.8) | 1223 (35.6) | <0.001 |
| LVEF (%), median (Q1, Q3) | 58.0 (51.0,65.0) | 58.0 (51.0,64.8) | 0.135 |
| Impaired heart function | 3349 (27.4) | 1808 (25.9) | 0.025 |
| Impaired renal function | 1344 (11.1) | 749 (10.9) | 0.575 |
| Diagnosis |  |  |  |
| STEMI | 4932 (40.4) | 3331 (47.8) | <0.001 |
| NSTEMI | 2159 (17.7) | 1144 (16.4) |  |
| UA | 5122 (41.9) | 2498 (35.8) |  |
| Thrombolysis* | 1994 (40.4) | 1349 (40.5) | 0.948 |

BMI = body mass index; TIA= transient ischemic attack; HR= heart rate; SBP=systolic blood pressure; DBP=diastolic blood pressure; CPR= cardiopulmonary resuscitation; TC= total cholesterol; LDL-C = low-density lipoprotein cholesterol; LVEF = left ventricular ejection fraction; STEMI = ST elevation myocardial infarction; NSTEMI = non–ST-segment elevation myocardial; UA= unstable angina; DAPT = dual antiplatelet therapy; LMWH = low molecular weight heparin ; ACEI = angiotensin-converting enzyme inhibitors; ARB = angiotensin receptor blockers; eGFR = estimated glomerular filtration rate.

#, among MI patients; *, among STEMI patients.

**Figure S1.** Flow chart of study participants

Flow diagram of the selection process of patients with DAPT from the CPACS-3 (Clinical Pathways for Acute Coronary Syndromes Phase 3) study population for the bleeding risk analysis.


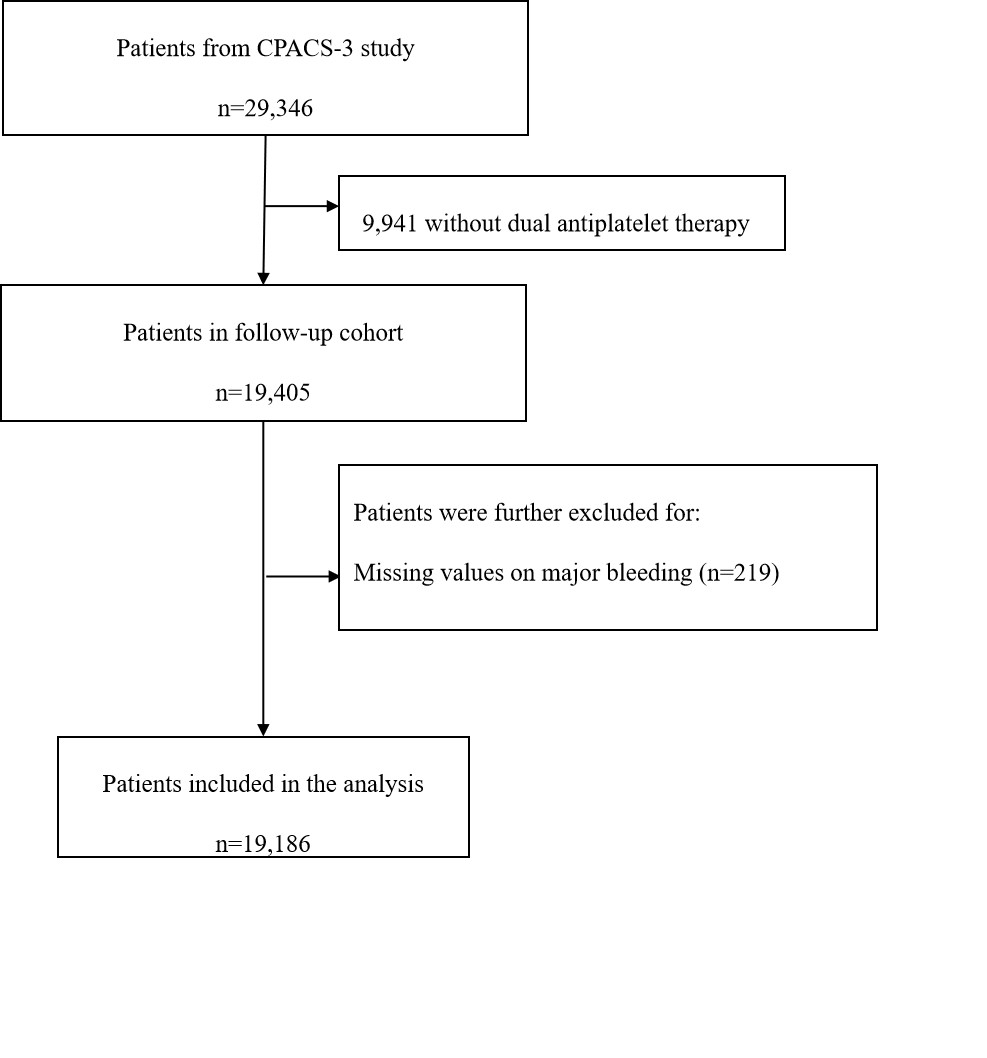


**Figure S2.** The distribution of the sites of major bleeding in patients with ACS.

The pie charge shows the number, percentage of patients with intracranial hemorrhage, gastrointestinal (GI) bleeding, and other or unclear bleeding. Other or unclear bleeding includes non-intracranial or non-GI bleeding, and cases where the site of bleeding is unknown.


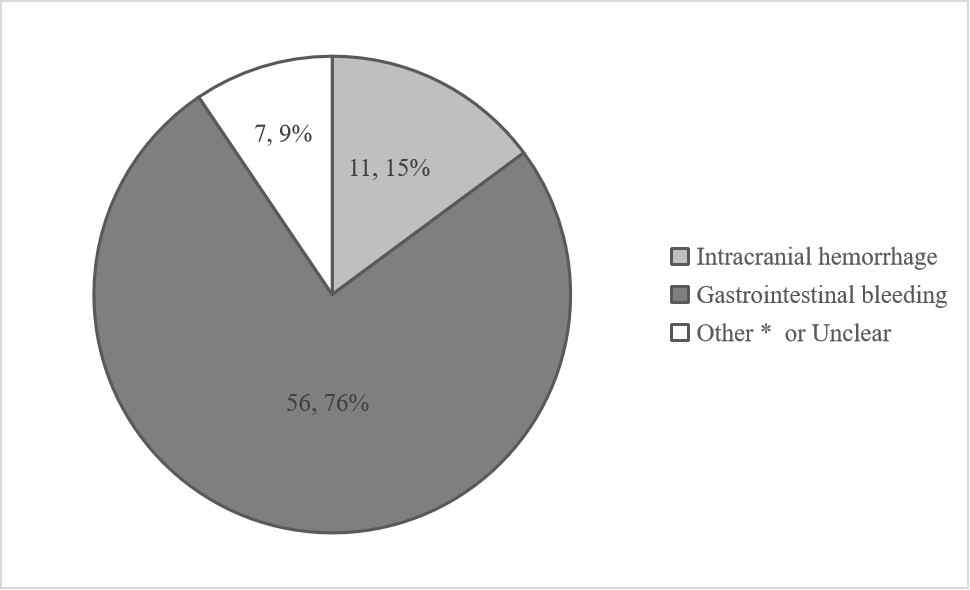


**Figure S3** ROC curves of CRUSADE score and the new model for predicting in-hospital major bleeding.


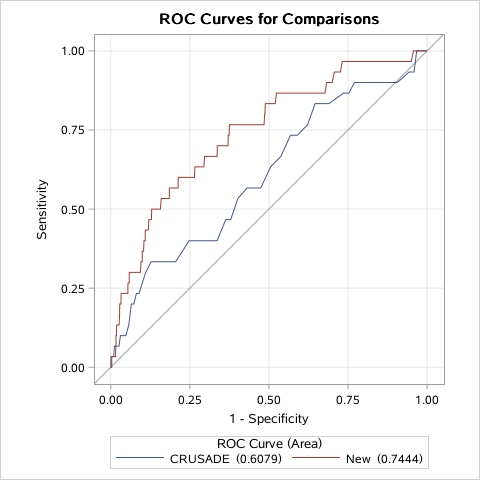

Supplement: Supplementary file 1 [file Data_Sheet_1.docx]
